# Coronal Sealing Capacity of Temporary Restorative Materials in Pediatric Dentistry: A Comparative Study

**DOI:** 10.5005/jp-journals-10005-1419

**Published:** 2017-06-01

**Authors:** Shabnam Milani, Bahman Seraj, Alireza Heidari, Atousa Mirdamadi, Mahdi Shahrabi

**Affiliations:** 1 Resident, Department of Pedodontics, Dentistry School of Tehran University of Medical Sciences, Tehran, Islamic Republic of Iran; 2 Associate Professor, Department of Pedodontics, Dentistry School of Tehran University of Medical Sciences, Tehran, Islamic Republic of Iran; 3 Assistant Professor, Department of Pedodontics, Dentistry School of Tehran University of Medical Sciences, Tehran, Islamic Republic of Iran; 4 Ex-Postgraduate Student, Department of Pedodontics, Dentistry School of Tehran University of Medical Sciences, Tehran, Islamic Republic of Iran; 5 Associate Professor, Department of Pedodontics, Dentistry School of Tehran University of Medical Sciences, Tehran, Islamic Republic of Iran

**Keywords:** Dye penetration method, Microleakage, Temporary restorative materials.

## Abstract

**Aims:**

The aim of this *in vitro* study is to compare the coronal microleakage of three common temporary restorative materials, namely Coltosol, Compoglass, and Zonalin, used in pediatric dentistry after endodontic treatment at different time intervals (1 week, 1, and 2 months) using dye penetration.

**Materials and methods:**

Access cavities were prepared in 72 intact extracted premolar teeth. The samples were divided into three groups (n = 24) and filled with Coltosol, Compoglass, or Zonalin. After thermal cycling for 500 cycles (5-55°C), the teeth were immersed in 1% methylene blue dye at 37°C for 1 week (n = 8), 1 month (n = 8), and 2 months (n = 8). The samples were sectioned buccolingually, and the linear depth of dye penetration was measured using a stereomicroscope at 16 × magnification. The data were analyzed using Kruskal-Wallis test.

**Results:**

There were no significant differences in the micro-leakage values of Coltosol and Zonalin or Zonalin and Com-poglass groups at 1 week (p > 0.05) or 1 month (p > 0.05) intervals, but a significant difference was noted between Coltosol and Compoglass groups (p < 0.01); Coltosol provided a more favorable coronal seal. No significant difference was found among the experimental groups at the 2-month interval (p > 0.05).

**Conclusion:**

At 1 week or 1 month of use, Coltosol showed better coronal seal. At 2 months, there was no significant difference apparent between the groups. A longer time lapse was associated with an increased likelihood of microleakage.

**How to cite this article:**

Milani S, Seraj B, Heidari A, Mirdamadi A, Shahrabi M. Coronal Sealing Capacity of Temporary Restorative Materials in Pediatric Dentistry: A Comparative Study. Int J Clin Pediatr Dent 2017;10(2):115-118.

## INTRODUCTION

Bacterial infection has been regarded as the most common cause of pulpal and periradicular disease.^1,2^ Therefore, the main goal in endodontic treatment is complete elimination of bacteria and preservation of the tooth in an uninfected environment feasible by preventing bacterial penetration during and after the treatment procedure.^3^ In many cases, the clinician is not able to complete the treatment with a permanent restoration in one session because of clinical complications or patient/clinician-related factors. Therefore, multiple sessions are inevitable.

Microleakage is defined as the leakage of microorganisms and toxins through the interface between the restoration and the walls of the cavity.^4^ Temporary restorative materials used between sessions of an endodontic procedure should have an acceptable sealing ability to prevent marginal leakage of fluids, debris, and microorganisms from the oral environment into the root canal system and restrain the degradation of therapeutic materials placed in the pulp chamber.^1,2^ Coronal leakage compromises the outcome of nonsurgical endodontic treatment.^5^ The quality of the coronal seal is as critical as the apical seal of the root canal filling for periapical health after root canal therapy.^6^

There are a variety of temporary restorative materials with different compositions, setting, and microstructure.^7^

Coltosol is a noneugenol, zinc oxide/zinc sulfate-based, self-setting, and single-component cement, which is used as a temporary filling material. Coltosol hardens by water absorption correlated with 17 to 20% hygroscopic expansion.^8^

Compoglass belongs to the compomer group.^9^ Com-pomer is a resin-based and polyacid-modified composite in which the ingredients are derived from composite and glass. Compoglass basically consists of bisphenol A glycidyl methacrylate , modified monomers, and fluoride-releasing fillers. It is manufactured as a single-component, light-cured material.^9-12^

Zonalin is a polymer-modified zinc oxide eugenol reinforced with 20 to 40% polymethyl methacrylate by weight and is manufactured as a powder and a liquid counterpart.^13^

There are various methods for assessing the coronal sealing ability of restorative materials, namely fluid infiltration, bacterial leakage, dye penetration, and dye extraction.^14,15^ In fluid infiltration, the sealing ability is evaluated by air bubble displacement in a capillary tube.^15^ An advantage to be pointed out is its quantitative assessment and possibility of performing longitudinal studies. On the contrary, nominal values are very low; thus, the actual leakage route is often obscure. It may also show false interfacial leakage values because of leakage through substrate itself.^16^

The method of investigation of bacterial leakage technique is variable by utilizing different bacterial strains.^15^ The studies are mostly qualitative as penetration of bacteria may lead to productivity and turbidity.^17,18^

In the dye extraction method, the teeth are immersed in an acidic environment capable of releasing the dye from the interface; the optical density of the solution is measured by a spectrophotometer.^15^ It is obvious that this method requires special devices.

In dye penetration, various dyes can be used, such as eosin, methylene blue, and black India ink. Many studies have used methylene blue as the dye substance as it is inexpensive, easy to manipulate, and has high degree of stainability and low molecular weight (even lower than bacterial toxins).^19^ This technique is widely used in leakage assessment studies because of its technical simplicity also.^20^

The aim of this *in vitro* study was to compare the sealing ability of three common temporary restorative materials used in pediatric dentistry after endodontic procedures namely Zonalin (Dental Products Ltd, England), Compoglass (Vivadent, USA), and Coltosol (Coltene, Switzerland) at different time periods via dye penetration.

## MATERIALS AND METHODS

This experimental study was conducted on 72 intact extracted premolars. The teeth were kept in 1% hypo-chlorite after extraction. After 3 days, the surfaces were cleaned using a scaler and the samples were immersed in normal saline until the time of experimentation. The samples were distributed into three main groups of 24 teeth, and each group was also divided into three subgroups of eight samples. The access cavity was prepared with a high-speed diamond fissure bur, and the contents of the pulp chamber were removed via an excavator. A cotton pellet was placed in the pulp chamber such that 4 mm space was left for placing the restorative material.

The first group was filled with Zonalin and it was condensed via a wet cotton pellet. In the second group, after applying a primer, Compoglass was placed according to the manufacturer’s instructions using a layering technique and was cured subsequently. The third group was filled with Coltosol. All the samples were immersed in 37°C normal saline and allowed to set for 48 hours. A 30-minute period was considered for complete drying of surfaces at room temperature. Thermal cycling was performed for 500 cycles at 5 and 55°C with a dwell time of 30 seconds in each bath. After thermal cycling, the surfaces of the teeth (except for the occlusal table) were covered with two layers of nail varnish; allowing to dry for 30 minutes after application of each layer. The apex was also sealed with sticky wax to prevent dye leakage. The samples were immersed in melted wax, except for the occlusal table.

The first group of samples (n = 24, 8 samples from each group) was kept separately at 37°C in glass vials containing 1% methylene blue dye for 1 week. The vials were labeled with the name of the restorative material and the period of immersion. After 1 week, the samples were thoroughly rinsed with running water; the layers of wax and nail varnish were removed with a scalpel. The teeth were sectioned buccolingually using a surgical handpiece and a diamond disc. To evaluate the degree of leakage, the samples were analyzed under 16× stereomicroscope by an examiner at two different times. According to the degree of leakage, grading was performed as follows: Grade 0 = without dye penetration, grade I = dye penetration up to the dentinoenamel junction (DEJ), grade II = dye penetration from the DEJ to half of the pulp chamber, grade III = penetration of dye to more than half of the pulp chamber.

This procedure was performed for other groups as well and assessed at 1 and 2 months of dye immersion. The data were analyzed using Kruskal-Wallis test.

## RESULTS

According to the findings, Coltosol showed better results at 1 week and 1 month of immersion, followed by Zonalin and Compoglass. A significant difference was found only between Compoglass and Coltosol (p < 0.05).

At 2 months evaluation, Coltosol showed better sealing ability, followed by Zonalin and Compoglass, but statistical assessment did not reveal any significant difference among these materials at this time point. The degree of significance decreased with time and there was an increase in the amount of microleakage. Leakage values in the groups are shown in Tables 1 and 2.

## DISCUSSION

This *in vitro* experimental study was designed to compare coronal sealing ability of three common temporary restorative materials used in pediatric dentistry namely Zonalin, Coltosol, and Compoglass at three different time intervals (1 week, 1, and 2 months) via dye penetration.

**Table 1:** **Table 1:** Dye penetration scores of the experimental materials

		*Zonalin*						*Coltosol*						*Compoglass*					
*Dye score*		*1 week*		*1 month*		*2 months*		*1 week*		*1 month*		*2 months*		*1 week*		*1 month*		*2 months*	
0		0		0		0		0		0		0		0		0		0	
I		1		0		0		4		0		0		0		0		0	
II		3		3		2		4		7		5		3		2		1	
III		4		5		6		0		1		3		5		6		7	
Total number		8		8		8		8		8		8		8		8		8	

**Table 2:** **Table 2:** Statistic data of coronal microleakage of the experimental materials at three time intervals

*Time*		*Materials*		*Mean*		*SD*		*Median*		*Kruskal wallis*		*p-value*	
1 week		Zonalin		2.371		0.74		2.5		9.665		0.0084	
		Coltosol		1.5		0.53		1.5					
		Compoglass		2.62		0.51		3					
1 month		Zonalin		2.62		0.517		3		6.0708		0.0349	
		Coltosol		2.12		0.354		2					
		Compoglass		2.75		0.463		3					
2 months		Zonalin		2.75		0.462		3		4.672		0.0967	
		Coltosol		2.37		0.052		2					
		Compoglass		2.87		0.354		3					

SD: Standard deviation

According to the results, at 1 week and 1 month, Coltosol showed better sealing ability in comparison to Zonalin and Compoglass respectively. There was a significant difference between Compoglass and Coltosol at 1 week and 1 month; thus, we can conclude that Coltosol provides more favorable coronal sealing. Such a significant difference was not observed between Coltosol and Zonalin or Zonalin and Compoglass. Analyzing the data proved that the degree of significance decreased with time and the results were similar. This can be attributed to the effect of time and possibly the solubility of these materials (e.g., Zonalin and Coltosol). After 2 months of immersion, no difference was found between the materials.

Mohammadian and Jafarzadeh-Kashi^21^ compared the degree of microleakage of three temporary restorative materials, namely Zonalin, Cavizol, and Coltosol, by dye penetration technique in an *in vitro* study. The prepared samples were immersed in methylene blue dye for 24 hours, 1 week, and 4 weeks. According to the results, microleakage was more profound in Zonalin group compared with Coltosol. These findings were in concordance with the results of our study, although the technical procedures differed.

Shahi et al^7^ evaluated the coronal sealing ability of four temporary restorative materials, namely IRM, Coltosol, Zonalin, and Zamheri, by using dye penetration. In this study, the samples were immersed in 10% India ink for 72 hours after thermocycling. The lowest and the highest amount of leakage were seen in Zonalin and Coltosol groups respectively. The difference between the results of this study and our study can be related to dissimilarities between the methods of conduction.

Naseri et al^22^ also compared the coronal sealing capacity of Coltosol, Cavizol, and Zonalin with dye penetration. Zonalin had more leakage values than Cavizol and Coltosol, and the amount of leakage increased from the 1st day to the 4th week. Their findings support the results of the current study.

Uranga et al^23^ assessed the coronal sealing ability of four restorative materials, namely Fermit, Tetric, Dyract, and Cavit, by dye penetration after thermocycling. The results showed that Tetric (composite) and Dyract (com-pomer) more convincingly prevented microleakage in comparison to Cavit and Fermit.

The sealing ability of materials may be subject to change in the oral environment or in the long term. Further clinical and laboratory studies are required to simulate biological conditions and reassess the results.

## CONCLUSION

The microleakage scores of all materials increased with time. At 1 week and 1 month of application, Coltosol had significantly better sealing capacities. At 2 months, there was no significant difference among Zonalin, Coltosol, and Compoglass, and leakage increased in all samples.
